# Fasting-mimicking diet remodels gut microbiota and suppresses colorectal cancer progression

**DOI:** 10.1038/s41522-024-00520-w

**Published:** 2024-06-25

**Authors:** Man Luo, Qingyi Wang, Yong Sun, Yao Jiang, Qiwen Wang, Yanrou Gu, Zhefang Hu, Qianyi Chen, Jilei Xu, Shujie Chen, Tongyao Hou, Lijun Feng

**Affiliations:** 1 Department of Clinical Nutrition, Sir Run Run Shaw Hospital, Zhejiang University, Hangzhou, China; 2 Medical School of Zhejiang University, Hangzhou, China; 3 Department of Gastroenterology, Sir Run Run Shaw Hospital, Zhejiang University, Hangzhou, China; 4 Department of Gastroenterology, Second Affiliated Hospital of Zhejiang University School of Medicine, Hangzhou, China; 5 Wenzhou Medical University, Wenzhou, China

**Keywords:** Microbiome, Microbiota

## Abstract

The progression of colorectal cancer is closely associated with diet. Fasting-mimicking diet (FMD) is a promising type of dietary intervention that have beneficial effects in the prevention and treatment of various cancers. We investigated the therapeutic effect of 4-day FMD against colorectal cancer in mice through immune cell analysis, microbiota composition analysis and anti-PD-1 treatment. These FMD cycles effectively suppressed colorectal cancer growth, reduced cell proliferation and angiogenesis, increased tumor-infiltration lymphocytes especially CD8^+^T cells. FMD stimulated protective gut microbiota, especially *Lactobacillus*. Supplementation of *Lactobacillus johnsonii* induced similar results as FMD intervention, which also suppressed tumor growth and increased CD45^+^ and CD8^+^ T cells. Additionally, FMD synthesizing with anti-PD-1 therapy effectively inhibited CRC progression. These findings suggest that *Lactobacillus. johnsonii* is necessary for the anticancer process of FMD in CRC. FMD through its effects on both gut microbiota and immune system, effectively suppressed colorectal cancer progression in mouse model.

## Introduction

Colorectal cancer (CRC) is a prevalent malignancy of the digestive system. According to Colorectal cancer statistics 2023^1^, CRC is the third most commonly diagnosed cancer worldwide, and the third leading cause of cancer deaths. The etiology of CRC involves both genetic and environmental factors. Modifiable lifestyle factors, especially dietary patterns are highly associated with the occurrence and progression of CRC^2^.

Tumor cells require elevated energy and nutrients for their rapid growth. Indeed, reduction in certain nutrients effectively regulates their proliferation. Calorie restriction or water-only fasting, promotes anti-tumor effect in various cancers, however, its clinical application faces challenges due to low adherence and acceptability^3^. Recently, A plant-based, calorie restricted fasting-mimicking diet (FMD) has been developed to stimulate similar physiological responses of fasting, while providing calorie and nutrients, thus, more feasible for patients to adhere^4^. Fasting or FMD suppresses the progression of colorectal cancer, characterized by heightened cell apoptosis and suppression of aerobic glycolysis^5–7^. Beyond its role in modulating tumor cell metabolism, FMD is also recognized as a promising anti-cancer agent due to its potential to reshape the immune response in cancer^6,8,9^. Recently, FMD have shown to boost antitumor immune responses by inhibiting the class switching of B cells to IgA, thereby stimulating antitumor immunity and suppressing colorectal cancer progression^10^. In various tumor-bearing mouse models, combining FMD with other treatments such as chemotherapy, immunotherapy therapy, exhibited enhanced, synergistic therapeutic effects^11,12^. FMD combining with chemotherapy elevates level of common lymphoid progenitor cells and cytotoxic CD8^+^ T cells in bone marrow, which significantly impeded the progression of breast and melanoma cancers^11^. However, the comprehensive understanding of the anti-cancer immune responses elicited by FMD and the effect of their integration with immunotherapy in the treatment of colorectal cancer remains unclear.

Gut microbiota as a major regulator for immune reactions, responds rapidly to dietary change^13^. Fasting can influence the gut microbiota’s composition and function, enhancing its beneficial interaction with the host to support homeostatic bacterial balance and boost immunity^14^. For example, time-restricted fasting and intermittent energy restriction attenuate colitis by reducing harmful bacteria, and enhancing short-chain fatty acid generating bacteria including *Lactobacillus, Rikenellaceae*. etc.^15^. Intermittent fasting enriched the gut bacteria diversity, with increased abundance of *Bacteroidaceae, Lactobacillaceae*, and *Prevotellaceae*, as well as enhancing microbial pathways related to antioxidant metabolism^16^. Currently, the only study focus on the gut microbiota change following FMD is on inflammatory bowel disease (IBD). It shows that fecal microbiota transplantation (FMT) from FMD-treated mice reduced the inflammation and reverse colon shortening of inflammatory bowel disease^17^. However, the interplay between FMD, the gut microbiota, and the immune system’s response to colorectal cancer is not yet fully understood.

This study aims to investigate the impact of cyclic FMD on the immune cell profile and gut microbiota composition in CRC mouse model, as well as to assess the synergistic effects of FMD combined with immunotherapy in enhancing CRC treatment. We expect that FMD would modulate tumor environment by enhancing antitumor immunity and promoting beneficial gut microbiota, and improve the effectiveness of immunotherapy in CRC.

## Result

### FMD suppresses colorectal tumorigenesis

**Fig. 1:**
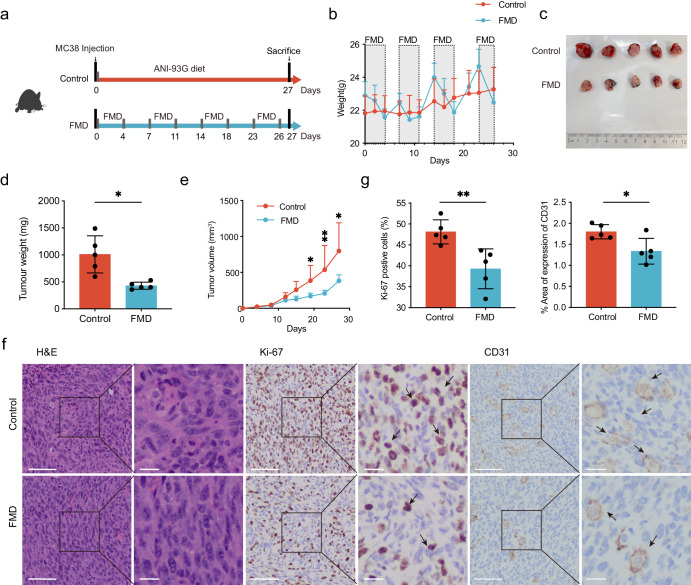
FMD suppressed tumor growth in subcutaneous tumor model. **a** Schematic diagram of FMD feeding regimen. MC38 cells were injected subcutaneously in the same day as FMD started, defined as Day 0. Mice underwent 4 cycles of FMD and sacrificed at Day 27. **b** Average body weight change during the study timeframe. **c** Image of dissected tumors at day of sacrifice. Upper: control group; lower: FMD group. **d** Tumor weight on day of sacrifice. **e** Average tumor volume measured after MC38 cells injection. **f** Representative images of H&E staining, immunohistochemistry (IHC) staining of Ki67 and CD31 in tumor tissue (black arrows indicating the positive staining of Ki67 and CD31). Scale bar: 100μm (left); 25μm (right). **g** Quantification of Ki67 positive cells and percentage area of CD31 expression. Two slices were prepared for each tumor tissue, 5 visions were randomly selected from each tissue for cell counting. Unpaired t test. Control group, *n* = 5; FMD group, *n* = 5. Data are shown in mean ± SD. * *p* < 0.05, ** *p* < 0.01.

To investigate the effect of FMD on colorectal cancer, subcutaneous tumor model of CRC was established (Fig. 1a). FMD was administrated by 4 days of FMD diet followed by 3 days of unrestricted normal diet. The reduction in body weight were within 10% during FMD cycles and regained quickly to even exceeding pre-fasting weight (Fig. 1b). We observed that FMD delayed CRC tumor growth and progression (Fig. 1c). Tumor weight was 57% lower in mice fed with FMD than those with normal diet, and the growth of tumor volume is slower in FMD group (Fig. 1d, e). H&E staining showed the structure of tumor tissues (Fig. 1f). Major reductions in the number of Ki67 positive cell and CD31 were observed after FMD cycles (Fig. 1f, g), but no difference were found in cell apoptosis (Supplementary Fig. 1), indicating that FMD effectively inhibit tumor cell proliferation without necessarily increasing cell apoptosis.

### FMD triggers protective immune cell response in tumor tissue and PBMCs

**Fig. 2:**
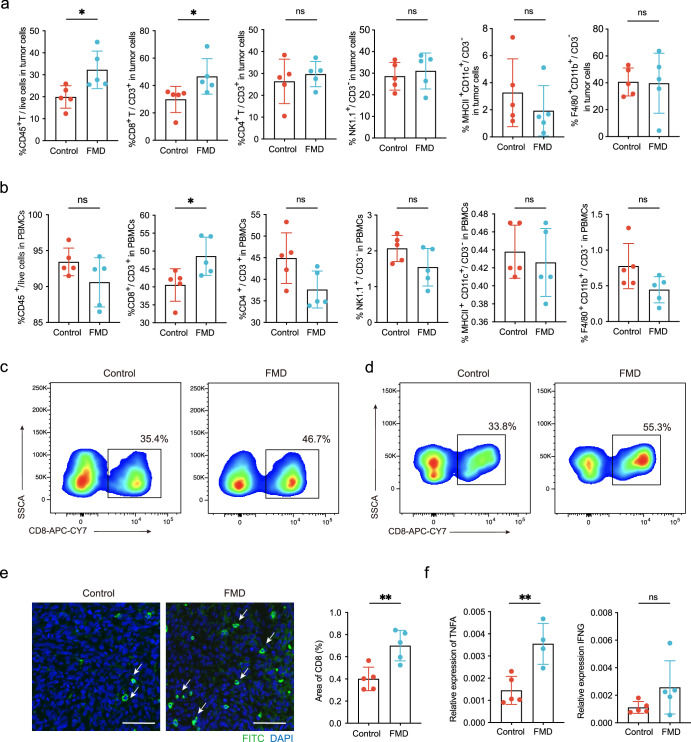
FMD increased CD8^+^ T cells in tumor cells and PBMCs. **a** Relative levels of CD45^+^ cells, CD8^+^ T cells, CD4^+^ T cells, NK cells, DC cells and macrophage in tumor tissue analyzed by multi-color flow cytometry. Mann–Whitney *U*-test for CD8^+^ T cells, unpaired t test for others. **b** Relative levels of immune cells in PBMCs. **c** Flow cytometry representation of CD8^+^ T cells in tumor cells. **d** Flow cytometry representation of CD8^+^ T cells in PBMCs. **e** Representative image of immunofluorescence staining of CD8 in tumor tissues (Left). The white arrow represents CD8 positively stained cells. Scale bar, 50 μm. The percentage area of CD8 expression was quantified by counting the average of three distinct fields in each sample (Right). **f** Relative expression of TNFA and IFNG in tumor tissues, one outlier of TNFA expression in FMD group was excluded by outlier identifier in Prism. Unpaired t test. Control group, *n* = 5; FMD group, *n* = 5. Data are shown in mean ± SD. * *p* < 0.05, **p < 0.01.

The immune system is crucial in cancer regulation and treatment response. To determine the effect of FMD on immune responses, we analyzed the expression of immune cells including CD45^+^, CD4^+^T cell, CD8^+^ T cell, macrophage, natural kill (NK) cell and dendritic cell (DC) using the gating strategy shown in Supplementary Fig. 2. No significant difference was found in macrophage, NK cell and DC cell (Fig. 2a, b). Higher tumor infiltrating lymphocytes were observed in the tumor cells of mice after four FMD cycles, represented by 12.3% increase in the percentage of CD45^+^ cells (Fig. 2a). The percentage of tumor infiltrating CD8^+^ T cells in CD3^+^ cells was 17% and 8% higher in both peripheral blood mononuclear cells (PBMCs) and tumor tissue of FMD group compared to normal diet group (Fig. 2b–d). The immunofluorescence analysis confirmed this result (Fig. 2e). We also analyzed the relative expression of CD8^+^ T cell activation marker tumor necrosis factor alpha (TNFA) and interferon-gamma (IFNG) in tumor tissue, the average relative expression of TNFA and IFNG in FMD group is 1.45 times and 1.3 times higher than control group (Fig. 2f).

### FMD reshapes the microbial community

**Fig. 3:**
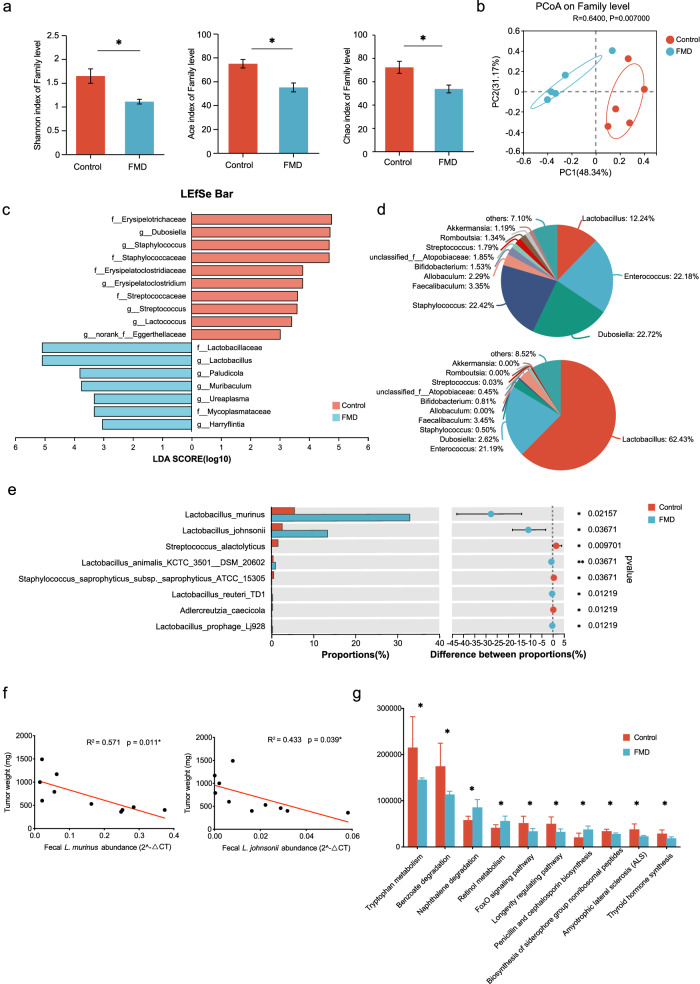
FMD changed gut microbiota composition. **a** α-diversity of the gut microbiome, represented by the Shannon, Ace and Chao diversity index of control and FMD groups on family level. **b** β-diversity of the gut microbiome shown by the principal coordinate analysis (PCoA) plot on family level. **c** Differences in bacterial taxa by Linear discriminant analysis Effect Size (LEfSe) analysis between control and FMD groups. Only taxa with a significant LDA > 3 were presented. **d** Pie graph showing the composition of microbial on genus level in fecal samples from the control and FMD groups. Upper: control group; lower: FMD group. **e** Significant alterations in control and FMD groups on species level. **f** Linear correlation of relative abundance of *L. murinus* and *L. johnsonii* and tumor weight. **g** PICRUSt2 KEGG prediction of microbial functions from 16S rRNA, showing the top 10 most abundant pathways at Level 3 differentially expressed pathways. Control group, *n* = 5; FMD group, *n* = 5. Data are shown in mean ± SD. Unpaired t test. *p < 0.05, **p < 0.01, ***p < 0.001.

Gut microbiota responds rapidly to dietary change. To explore the effect of FMD on gut microbiota change, we collected the fecal samples from mice at the end of the last FMD cycle, and performed 16S rRNA sequencing on fecal sample. Four cycle of FMD displayed major changes in the gut microbial composition of mice. The alpha-diversity (Shannon index, Ace index and Chao index) at family level (Fig. 3a) reduced after FMD, beta diversity was shown by principal coordinates analysis (PCoA) calculated by Bray-Curtis metric, marked separation was found between control and FMD group (*p* = 0.007), indicating the significant difference in microbiol communities (Fig. 3b).

Using the LEfSe algorithm, we identified notable taxonomic differences between groups. *Lactobacillaceae* and *Lactobacillus*, as protective microbiol strains that involves in T cell regulation^18^, increased by approximately 5-fold in FMD group (Fig. 3c, Supplementary Fig. 3a); while *Erysipelotrichaceae*, a microbiol family associated with inflammation-related disorders and enriched in both CRC cancer patients and experimental models^19,20^, reduced by 4.9-fold in FMD group (Fig. 3c, Supplementary Fig. 3a, b). At Genus level, *Staphylococcus* (22.4%), *Dubosiella* (22.7%) and *Enterococcus* (22.2%) prevail in control group; while *Lactobacillus* (62.4%) dominated in FMD group, the proportion of *Enterococcus* (21.19%) remained similar, and all other bacteria reduced (Fig. 3d, Supplementary Fig. 3c). At species level, the relative abundance of *Lactobacillus murinus* increased 6-fold and *Lactobacillus johnsonii* increased 5-fold in FMD group, the same results were found by qPCR verification of fecal samples (Fig. 3e, Supplementary Fig. 3d). When conducting the correlation analysis between these two predominant bacterial and tumor weight, we found that both *L. murinus* and *L. johnsonii* were significantly negatively correlated with tumor weight, indicating the beneficial effect of the two bacteria in tumorigenesis (Fig. 3f).

To further investigate the potential mechanisms of FMD-induced gut microbiota change on colorectal cancer carcinogenesis, we conducted PICRUSt2 analysis to predict the functions of microbial communities. The KEGG pathway level 3 prediction revealed 417 categories that were compared between control and FMD groups, with 28 of them showing differential expression between groups. Within the top 10 most abundant pathways identified, naphthalene degradation, retinol metabolism penicillin and cephalosporin biosynthesis were upregulated in FMD group, while tryptophan metabolism, benzoate degradation, Foxo signaling pathway, longevity regulating pathway, biosynthesis of siderophore group nonibosomal peptides, amyotrophic lateral sclerosis (ALS) and thyroid hormone synthesis were downregulated in FMD group (Fig. 3g). By PICRUSt2 KEGG enzyme analysis, phosphoglycerate mutase (PGM), an important enzyme involved in the glycolysis^21^, is predicted to be the most abundant enzyme among all the differentially expressed enzymes between groups (Supplementary Fig. 3e).

### *L. johnsonii* suppresses colorectal tumorigenesis

**Fig. 4:**
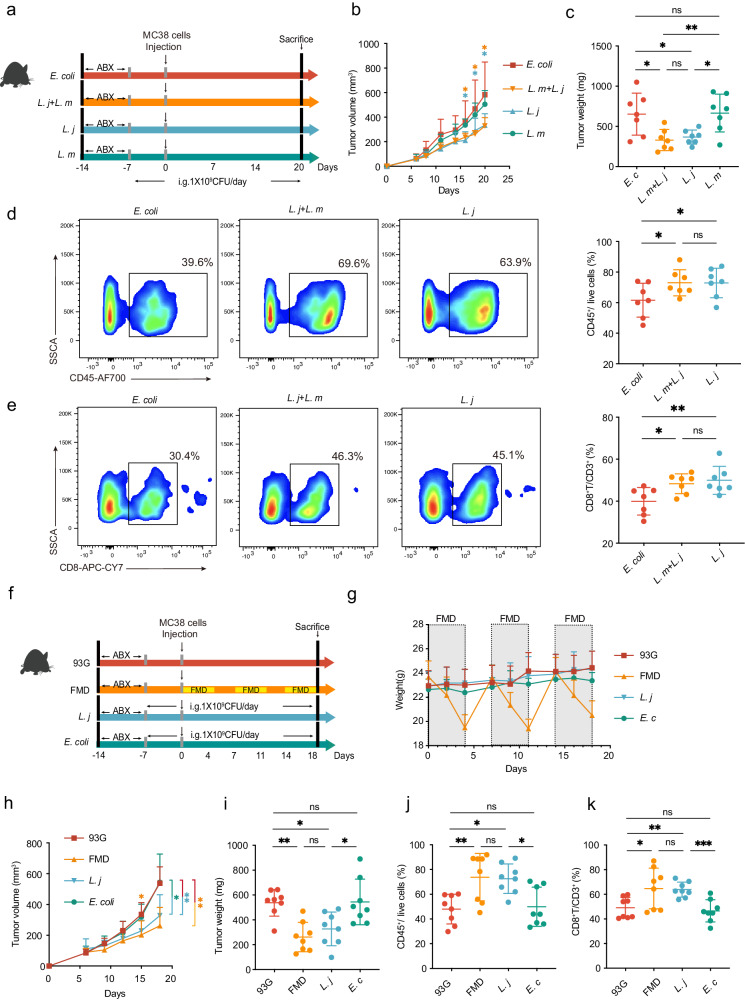
*L. johnsonii* induced similar anti-tumor effect as FMD in CRC mouse model. **a** Schematic diagram of oral gavage regimen of bacteria. *E. coli* group: *n* = 7; *L. m* + *L. j* group: *n* = 7; *L. j* group: *n* = 7; *L. m* group: *n* = 7. **b** Average tumor volume measured after MC38 cells injection. yellow asterisk: *E. c*. vs. *L. m* + *L. j*; blue asterisk: *E. c*. vs. *L. j*. Welch ANOVA test. **c** Tumor weight on day of sacrifice. One-way ANOVA. **d** Flow cytometry representation and cell counts of CD45^+^ cells in tumor tissue in three groups. One-way ANOVA. **e** Flow cytometry representation and cell counts of CD8^+^ T cells in tumor tissue. One-way ANOVA. **f** Schematic diagram of FMD and oral gavage of bacteria. 93 G group: *n* = 8; FMD group: *n* = 8; *L. j* group: *n* = 8; *E. c* group: *n* = 8. **g** Average body weight change during the study timeframe. **h** Average tumor volume. Yellow asterisk: 93 G vs. FMD; blue asterisk: 93 G vs. *L. j*; green asterisk: *E. c* vs. *L. j*. One-way ANOVA. **i** Tumor weight on day of sacrifice. One-way ANOVA. **j** Percentage of CD45^+^ cells in live cells in tumor tissue. One-way ANOVA. **k** Percentage of CD8^+^ T cells in CD3^+^ cells tumor tissue. Welch ANOVA test. Data are shown in mean ± SD. *p < 0.05, **p < 0.01, ***p < 0.001.

To verify whether *Lactobacillus* works in the inhibition of CRC progression, the bacteria strains with the highest differences between groups (*L. murinus* and *L. johnsonii*) was administered by oral gavage to CRC mouse models. The control group received oral gavage of *Escherichia. coli* (Fig. 4a), a non-pathogenic bacterial strain, and the intervention groups were treated with *L. murinus, L. johnsonii*, and an additional group of *L. murinus* mixed with *L. johnsonii* to verify the effect of microbial consortium (Fig. 4a). By three weeks of oral gavage, *L. j* and *L. j* + *L. m* consortium displayed a significant reduction in tumor growth, while *L. m* group did not show significant difference (Fig. 4b, c). To further investigate whether the bacteria cause changes in immune response, we monitored changes in tumor tissue. *L. m* group was not analyzed due to non-significant changes in tumor sizes, all other groups were assessed through flow cytometry. CD45^+^ cells were 18% higher in *L. j* and *L. j* + *L. m* group compared to control group (Fig. 4d); CD8^+^ T cells were approximately 10% higher in *L. j* and *L. j* + *L. m* group compared to control group, while no difference were found between *L. j* and *L. j* + *L. m* group (Fig. 4e). These results show that *L. johnsonii* plays the key role in suppressing tumor growth, *L. murinus* itself is ineffective, and the *L. j* + *L. m* microbial consortium is unable to enhance the anti-tumor effect of *L. j*.

Next, we compared the effect of *L. johnsonii* gavage and FMD treatment alone on CRC model (Fig. 4f). Since the “binge-like” eating behavior observed in the previous experiment caused enormous fluctuation in weight (Fig. 1b), which may associate with metabolic impairments^22^, we limited the feeding amount to the same level as the average daily consumption of control group. Mice in the FMD group lost ~20% of body weight during each energy restriction period and regained back quickly to normal weight at re-feeding period (Fig. 4g). By comparing to mice fed with normal diet and *E. coli* gavage, both *L. j* and FMD treatments markedly retarded tumor growth (Fig. 4h, i), as well as inducing increases anti-cancer immune responses, in particular in CD45^+^ and CD8^+^ T cells (Fig. 4j, k). No significant differences were found between the two groups, indicating that *L. j* exhibited similar anti-cancer effects as FMD.

To explore whether *Lactobacillus* strain is necessary in the anticancer effect of FMD, we performed vancomycin depletion experiment to clear *Lactobacillus* and *L. johnsonii* (Supplementary Fig. 4a, b)^23,24^. Substantial reduction of *Lactobacillus* and *L. johnsonii* were confirmed after 5-day vancomycin treatment. In mice treated with FMD, the depletion of *Lactobacillus* and *L. johnsonii* led to a significant increase in tumor growth and tumor weight (Supplementary Fig. 4c–e), the anti-tumor effect of FMD was attenuated, indicating that the delaying effects of FMD on CRC growth was relied on *Lactobacillus*, especially on *L. johnsonii* species.

### Synergistic effect of FMD and PD-1 blockade on CRC

**Fig. 5:**
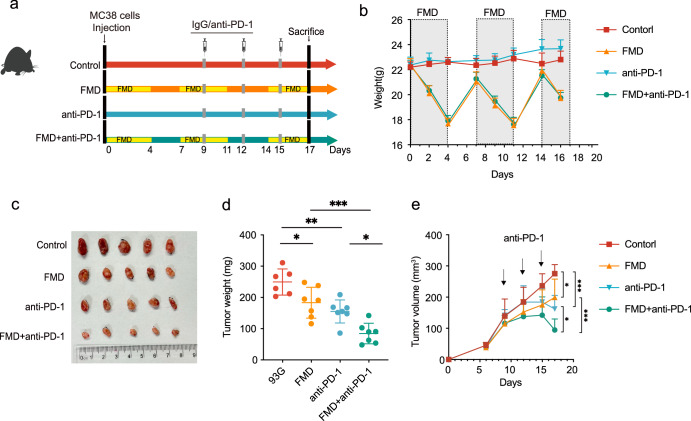
The combined treatment of FMD and anti-PD-1 synergistically retarded tumor growth in subcutaneous tumor model. **a** Schematic diagram of anti-PD-1 treatment of mice. MC38 cells were injected subcutaneously in the same day as FMD started. Mice in control and FMD groups were treated with IgG isotype, mice in anti-PD-1 and FMD+ anti-PD-1 groups were treated with monoclonal antibody at day 9, 12 and 15. Mice were euthanized at day 17. **b** Average body weight change during the study timeframe. **c** Image of dissected tumors. **d** Tumor weight on day of sacrifice. One-way ANOVA. **e** Average tumor volume measured after MC38 cells injection. Control: *n* = 6; FMD *n* = 7; anti-PD-1: *n* = 7; FMD+anti-PD-1: *n* = 7. Data are shown in mean ± SD. One-way ANOVA test. *p < 0.05, **p < 0.01, ***p < 0.001.

Due to the observed association between FMD and T-cell response to tumors, we further explore the effect of FMD combining with other treatment related to immune regulation. Here, we tested the synergistic effect of FMD and anti-PD-1 antibody on the growth of subcutaneous CRC tumor. Mice were fed with either control diet or FMD, the PD-1 blockade was administered every three days (Fig. 5a). Mice in the FMD group lost ~20% of body weight during each energy restriction period and regained back quickly to almost normal weight at re-feeding period (Fig. 5b). In both standard diet and FMD conditions, anti-PD-1 therapy has demonstrated efficacy in decelerating tumor growth (Fig. 5c–e). When combining FMD and PD-1 blockage, the tumor size, weight and volume significantly reduced compared to anti-PD-1 treatment alone, indicating that the synergistic application may enhance anti-tumor responses (Fig. 5c–e).

## Discussion

In this study, we demonstrate that FMD cycles has a positive effect on suppressing colorectal cancer development. Specifically, FMD lead to a reduction in tumor growth, tumor weight, and a decrease in the expression of the Ki67 and CD31, which is often associated with cancer cell proliferation and blood vessel formation. FMD changes tumor immune microenvironment by increasing CD45^+^ cells in tumor cells, as well as CD8^+^ T cells in both tumor cells and PBMCs, indicating the increased immune cell infiltration and the cancer-fighting ability of FMD. FMD alters gut microbiota composition, increases beneficial gut microbiota, specifically *Lactobacillaceae* family. Within this family, *Lactobacillus murinus* and *Lactobacillus johnsonii* increase most significantly. Previous studies demonstrated the important role of *Lactobacillus* in T cell regulation^25^. To explore whether *Lactobacillus* is involved in the inhibition process of tumor development by FMD, we administered oral gavage of *L. murinus* and *L. johnsonii*, and found that *L. johnsonii* exerts an anti-tumor effect that is similar to the effect of FMD, elimination of *Lactobacillus* and *L. johnsonii* weakened the anti-tumor effects of FMD, indicating the tumor suppressing effect of FMD may rely on *L. johnsonii*. Additionally, we demonstrated that FMD synthesizing with anti-PD-1 therapy effectively inhibited CRC progression.

Compiling evidence have shown the profound effects of fasting or fasting-like conditions on colorectal cancer prevention^26,27^. Our results are consistent with previous studies that fasting inhibited cell proliferation^6^; FMD cycles significantly postponed tumor progression and decelerated tumor growth in the CT26 subcutaneous tumor model^28^. The development of cancer induces a range of functional and compositional alterations of immune system^29^. Our study elucidates how FMD changes the immune microenvironment in colorectal cancer. We demonstrate the increase of CD8^+^ T cells in both tumor cells and PBMCs in CRC. The expansion of CD8^+^ cytotoxic T lymphocytes is able to stimulate the adaptive immune reaction against cancer, release cytotoxic molecules that induces apoptosis in cancerous cells^30^. In consistent with our results, Di Biase et al.^11^ reported an increase in circulating CD8^+^ lymphocytes in blood and tumor tissue of BALB/c mice with breast cancer after three FMD cycles; short-term starvation stimulates tumor-infiltrating CD8 T cells in lung cancer^31^. Clinical research has also confirmed this finding, a study involving 101 patients with different cancer types who received FMD treatment, showed that FMD reshapes systemic and tumor-associated anti-tumor immune responses including recruiting activated CD8^+^ T cells^8^. The immune system modulation by FMD found in our study, aligning with earlier work, suggesting that FMD can modulate the immune microenvironment to boost CD8^+^ T cell activity.

Gut microbiota responds quickly to changes in diet structure. In this study, we show that FMD alters the microbiota structure, in particular, increases the abundance of probiotics *Lactobacillus*. Similar results have been reported in an FMD-intervened IBD model, transplant of *Lactobacillus* effectively reversed DSS-induced colon shortening^17^. The increase in *Lactobacillus* after FMD cycles is possibly due to the plant-based, high fiber ingredients of FMD, including oligofructoses, fructo-oligosacharides, and galactomannan, extracted from vegetables can promote the proliferation of beneficial probiotic strains^17^. Besides, the polyphenol content derived from plant-based food has been found to increase the abundance of *Lactobacillus*^25^. At species level, the abundance of *L. murinus* and *L. johnsonii* increased significantly with FMD. *L. johnsonii* and *L. murinus* are proven probiotics with broad anti-inflammatory properties, *L. johnsonii* act as immune-modulator to alleviate ulcerative colitis, while *L. murinus* have beneficial role in enterocolitis^32–34^. Apart from the individual functional attributes exhibited by singular bacteria, the intricacies of collaborative microbial interactions have gained more attention. Microbial interactions may lead to mutual benefits through the exchange of metabolites, which may contribute to the enhancement of tumor microenvironment and immune responses^35^. Here, we expand this concept to *L. murinus* and *L. johnsonii*, exploring their potential roles as singular bacteria and in microbial combination. *L. johnsonii* demonstrates anticancer effects similar to FMD intervention, significantly delaying tumor growth and increasing CD8^+^ T cells. However, combining *L. johnsonii* with *L. murinus* did not enhance the anticancer effects, indicating that *L. johnsonii* may be the key species among the *Lactobacillus* genus that is enhanced by FMD, aiding FMD’s ability to suppress tumor growth. When depleting *Lactobacillus* and *L. johnsonii* with vancomycin, the effectiveness of FMD was attenuated. Thus, we propose that *Lactobacillus*, especially *L. johnsonii* species, is necessary for the anticancer process of FMD in CRC. Our investigation elucidates the function of gut microbiota in the context of FMD, demonstrating that the proliferation of beneficial probiotics like *Lactobacillus* is crucial in modulating the host’s immune and metabolic responses.

In tumor development, the combination of PD-1 and its corresponding ligand programmed death-ligand 1 (PD-L1) can lead to cytotoxic T cell exhaustion, which impairs the immune system’s ability to recognize and attack the cancer cells^36^. The inhibition of the binding between PD-1 and PD-L1 using monoclonal antibodies has demonstrated significant clinical efficacy in treating various types of cancer^31^. However, CRC has long been recognized as immunogenic and resistant to immunotherapy due to its low mutation burden and levels of immune cell infiltration^37^. Fasting-mimicking conditions enhance tumor immunogenicity and the presence of CD8^+^ T cells, which are the two essential prerequisites for the anti-tumor immune responses facilitated by PD-1/PD-L1 blockade^31^. Research have shown the synergistic effect of short-term starvation and anti-PD-1 immunotherapy suppressed the progression and metastasis of melanoma, breast and lung cancer^31,38^. Aligning with previous research demonstrating that FMD, either as a standalone treatment or combining with anti-PD-L1 therapies, substantially exceed immune checkpoint inhibitors alone in delaying melanoma progression in mice^39^. Our study support this notion and extended the content to colorectal cancer. We revealed that anti-PD-1 significantly slows tumor growth in mice treated with both standard diet and FMD; FMD combining with anti-PD-1 therapy is more effective than using either approach alone. These findings may offer an effective approach to improve the effectiveness of immunotherapy and the compliance of nutritional intervention in the treatment of colon cancer.

Collectively, our findings show that FMD suppresses CRC progression, increases immune responses, stimulates gut beneficial bacteria, especially *L. johnsonii*, which appears to be one of the key factors in the anticancer effect of FMD, and enhances the PD-1 blockade therapy in experimental models. Further investigation is warranted to explore the molecular mechanisms on how FMD interacts with the gut microbiome, particularly on the role of *Lactobacillus* in mediating immune responses. Well-designed clinical trials need to be conducted to assess the safety, tolerability, and efficacy of FMD as a dietary intervention in combination with standard CRC treatments, as well as immunotherapy. Although it cannot replace the standard cancer treatment, FMD may be used as complementary approaches to support overall health and well-being.

### Limitations to the study

There are some limitations to this study that need to be acknowledged. First, when using vancomycin to clear *Lactobacillus* and *L. j*, other gram-positive bacteria may be cleared as well. To avoid this, specific bacteriophages can be used to clear the target bacteria. Second, although preliminary KEGG pathway prediction analyses suggest a potential association with key enzymes in glycolysis, the downstream molecular mechanisms require further investigation. Additionally, the feasibility of FMD in clinical settings require confirmation by more clinical trials. The strict dietary regimen and low energy intake may induce adverse effects such as malnutrition, progressive weight loss, cachexia etc., especially in fragile individuals^14^. While previous clinical study has demonstrated the safety of FMD in human use^8^, ongoing monitoring and individualized dietary plans, meal quality and specific food items remain crucial to mitigate potential adverse effects.

## Methods

### Mouse models

Male C57BL/6 mice (6–8 weeks old) were purchased from Shanghai Laboratory Animal Center (SLAC), China. Mice were group-housed (4–5 mice/cage) under specific pathogen-free (SPF) conditions in barrier environment, with constant temperature and humidity, 12 h circadian rhythm and free access to water. Body weight were measured 2-3 times per week. Mice were euthanatized by CO_2_ inhalation, following cardiac puncture to ensure their euthanasia.

#### FMD + CRC model

Mice were randomly assigned to control and FMD group. Control group were fed with AIN-93G standard laboratory chow and FMD group were fed with 4-day regimen of FMD following 3-day unrestricted AIN-93G. 2 ×10^6^ MC 38 cells were injected subcutaneously under isoflurane anesthesia exposure in the same day as FMD started.

#### CRC+bacteria tumor model

All mice were fed with AIN-93G. Before bacterial administration, mice were orally supplemented with 100ul antibiotic (ABX) mixture (ampicillin, gentamicin, neomycin and metronidazole at 1 mg/ml; vancomycin at 0.5 mg/ml) for 7 days for initial microbiota consistency. The bacteria were applied by daily oral gavage. Group *E. coli* received 1 ×10^9^ CFU of *Escherichia coli MG1655* (*E. coli*) suspended in 200 μl PBS, Group *L. m* and group *L. j* received same amount of *Lactobacillus. murinus* (*L. murinus, L. m*) and *Lactobacillus. johnsonii* (*L. johnsonii, L. j*). Group *L. m* + *L. j* received a bacterial mix of *L. johnsonii* and *L. murinus* in a ratio of 1:1, oral gavage continued until mice sacrifice. For FMD + vancomycin model, mice were treated with 0.25 g/L vancomycin in drinking water 5 days before MC38 injection to clear *Lactobacillus* and *L. j*, and continued throughout the study period to maintain the low *Lactobacillus* level. 2 ×10^6^ MC 38 cells were injected subcutaneously under anesthesia conditions after pre-treatment of bacteria or antibiotics.

#### FMD+anti-PD-1 + CRC tumor model

Mice were randomly assigned to control, FMD, anti-PD-1 and FMD+anti-PD1 group. Mice in control and anti-PD-1 group were fed with AIN-93G, and mice in FMD and FMD + anti-PD-1 group were fed with 4-day regimen of FMD. 2 ×10^6^ MC38 cells were injected subcutaneously in the same day as FMD started. Mice were treated with IgG (Cat#Be0089; BioXcell, USA) or anti-PD-1 monoclonal antibody (100 ug/mouse, Cat# 0273, BioXCell, USA) at day 9, 12 and 15. Mice were euthanized at day 17.

### Animal diets

Animals in the control group was fed with AIN-93G standard animal chow (ReadyDietech, China) containing 15.8 kJ/g of energy. One cycle of FMD diet contains three components (ReadyDietech, China): Day 1 diet restricted 50% of normal energy intake (energy: 7.67 kJ/g; protein: 0.46 kJ/g; carbohydrate: 2.2 kJ/g; fat: 5 kJ/g), day 2–4 diet restricted 90% of normal energy intake (energy: 1.46 kJ/g; protein/fat: 0.01 kJ/g; carbohydrate: 1.47 kJ/g;), on day 5–7, mice were fed with standard animal chow. The feeding regimen was formed with several cycles of FMD diet.

### Ethics statement

Ethical approval was obtained from the Clinical Research Ethics Committee of Sir Run Run Shaw Hospital, Zhejiang University School of Medicine (ZJU20220379). The animal experiments conducted during the study followed the guidelines set by the Animal Experimentation Ethics Committee of Zhejiang University.

### Bacterial strain and cell cultural

The MC38 cells were purchased from Cell Resource Center, Institute of Basic Medicine, Chinese Academy of Medical Sciences (Cat No. 1101MOU-PUMC000523, Beijing, China). *L. murinus* and *L. johnsonii* were isolated from pig feces by the Zhejiang Academy of Agricultural Sciences. Their species level confirmation was achieved through 16S ribosomal RNA sequencing (V4 sequences). To cultivate the bacteria, De Man, Rogosa and Sharpe (MRS) Medium (HB0384-5, hopebio, China) were used, and the cultivation process took place for 24 h at 37 °C within an environment comprising 10% H_2_, 10% CO_2_, and 80% N_2_ using AW500SG anaerobic workstations (ELECTROTEK, England). As for the control, the nonpathogenic commensal intestinal bacteria, *Escherichia. coli* strain MG1655 (Biobw, China), were cultured in Luria-Bertani (LB) Medium (A507002 Sangon Biotech, China) at 37 °C. The final concentration of the cultures was adjusted to 1 × 10^9^ CFU/200 μl.

### Bacteria quantification and 16S rRNA sequencing

Bacterial DNA from mice fecal samples was extracted using TIANGEN stool kits (DP328-02, TIANGEN, Beijing). 16S rRNA sequencing was performed at Majorbio, China. Quantitative real-time PCR were used for *Lactobacillus*, *L. murinus* and *L. johnsonii* quantification. qPCR SYBR Green Master Mix (Cat No. 11198ES08; YEASEN, China), primers and template gDNA were used in triplicate for each reaction. The primer sets were shown in Supplementary table1.

### Histopathological analysis

The tumor tissues were fixed in formalin overnight at room temperature and subsequently embedded in paraffin. Sections of 5μm thickness were prepared for pathological examination and stained with hematoxylin and eosin (HE) staining. Immunohistochemistry was carried out on paraffin-embedded tissue sections using CD31 antibody (1:2000; Abcam Cat# ab182981, RRID: AB_2920881) and Ki67 antibody (1:400; Cell Signaling Technology Cat# 9129, RRID: AB_2687446). Immunofluorescence was performed by incubating paraffin-embedded tissues with CD8 antibody (1:1000; Abcam Cat# ab209775, RRID: AB_2860566). Images were collected with a positive fluorescence microscope. (Leica DM4000) and processed with ZEN image software.

### Flow cytometry analysis

The immune cells collected from tumor tissues and blood were processed. Blood were collected in K2 EDTA blood collection tube, and incubated in RBC (Red Blood Cell) lysis buffer with 5× the volume of blood for 15 min at room temperature. Blood were centrifuged at 800 × *g* for 10 min at 20 °C. After removing the supernatant, the pellet was resuspended in RPMI media (VWR, catalog #VWHRL0106-0500), and cell count was carried out with final dilution of 4 × 10^6^ cells per ml. Cells from tumor tissues and blood were stained by Fixable viability stain 510 (BD Biosciences Cat# 564406, RRID: AB_2869572) for 30 min. After termination, the following antibodies were used: CD45: Alexa Fluor 700 (BioLegend Cat# 103128, RRID:AB_493715); CD3: PECP-CY5.5 (BD Biosciences Cat# 551163, RRID:AB_394082); CD4: Brilliant Violet 605 (BioLegend Cat# 100548, RRID:AB_2563054); CD8: APC-CY7 (BD Biosciences Cat# 561967, RRID:AB_10893346); NK1.1: APC (BioLegend Cat# 108710, RRID:AB_313397); MHCII: PE (BD Biosciences Cat# 557000, RRID:AB_396546); CD11c: PE-CY7 (BioLegend Cat# 117318, RRID:AB_493568); CD103: FITC (BioLegend Cat# 121419, RRID:AB_10709438). Cells were fixed with fixation buffer (BioLegend, Cat# 422101) in dark for 30 min at 4 °C. Cell samples were transferred into tubes and analyzed by Flow Cytometer and FlowJo version10.8. In order to ensure comparability, a uniform sample size of 20,000 events was randomly selected from the individual cell subsets (CD45^+^ cells) of each group (control and FMD); 10,000 events were randomly selected for *E. coli*, *L. j* + *L. m* and *L. j* groups due to limited overall cell counts.

### Cell apoptosis

Cells were collected from tumor tissues. Cells were stained by Annexin V-FITC/PI cell apoptosis detection kit (Cat No. 40302ES50; Yeasen, China) as per manufacturer’s instruction. The cell apoptosis was analyzed by Flow Cytometer and FlowJo version10.8.

### Statistic analysis

Experimental results were analyzed and graphed using GraphPad 9.0 software. Flow cytometry results were analyzed with FlowJo 10.8. ImageJ 1.53 was used for quantitative analysis of immunofluorescence and immunohistochemistry staining images. The microbiome analyses were conducted utilizing the Majorbio Cloud online platform (Majorbio Bio-Pharm Technology Co. Ltd. Shanghai, China). Two-sided unpaired Student’s *t* tests (normal distribution) or Mann–Whitney *U*-test (non-normal distribution) were used for comparisons between two groups. Ordinary one-way ANOVA (normal distribution with equal variance) or Welch ANOVA tests (normal distribution with unequal variance), Kruskal–Wallis test (non-normal distribution) were used for comparisons among multiple groups. Linear correlation is used to measure the relationship between two variables. The specific statistical tests used were indicated in figure legend. The results were expressed as mean ± SD (standard deviation). *p* < 0.05 were defined statistically significant.

### Risk of bias

Mice were randomly assigned to groups and randomly housed in animal room to mitigate selection bias; All mice were group-housed under specific pathogen-free (SPF) conditions in barrier environment, with constant temperature and humidity to minimize the bias caused by environmental conditions. Dietary regimen and feeding time were consistent throughout the experiment to minimize the possible confounders such as variations in nutrient intake, circadian rhythm disruptions.

### Supplementary information

Supplementary information

## Data Availability

The 16S rRNA-seq raw data of this study is available in NCBI database under accession code PRJNA1104798. The corresponding author will provide access to other data sources and findings related to the study on reasonable request.
