# Supplementary material for: Fasting-mimicking diet remodels gut microbiota and suppresses colorectal cancer progression
Source: NPJ Biofilms Microbiomes. 2024 Jun 25;10:53. doi: 10.1038/s41522-024-00520-w (PMC11199600; doi:10.1038/s41522-024-00520-w)
Supplement: Supplementary file 1 — Supplementary information [file 41522_2024_520_MOESM1_ESM.pdf]

Supplementary data

qPCR	Forward primer	Reverse primer
Lactobacillus murinus	TCGAACGAAACTTCTTTATCACC	CGTTCGCCACTCAACTCTTT
Lactobacillus johnsonii	TCGAGCGAGCTTGCCTAGATGA	TCCGGACAACGCTTGCCACC
Lactobacillus	GAGGCAGCAGTAGGGAATCTTC	CCAGCGTTGCCACCTACGTA
Universal Eubacteria 16S	CGGCAA CGAGCGCAACCC	CCATTGTAGCACGTGTGTAG CC
Tumor necrosis factor alpha	CTGAACTTCGGGGTGATCGG	GGCTTGTCACTCGAATTTTGAGA
Interferon gamma	GCCACGGCACAGTCATTGA	TGCTGATGGCCTGATTGTCTT

Supplementary Table 1. Primers for bacteria and cytokines.

Supplementary Figure.

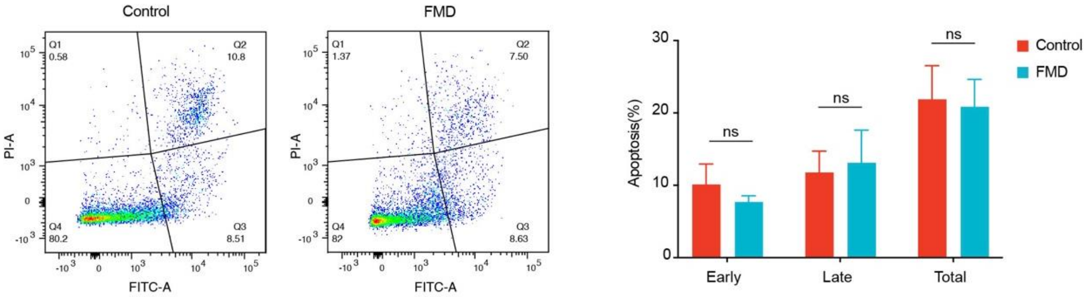

Supplementary Figure 1. Early, late, and total cell apoptosis of CRC tumor cells using Annexin V-FITC/PI staining. Control: n=5; FMD: n=5.

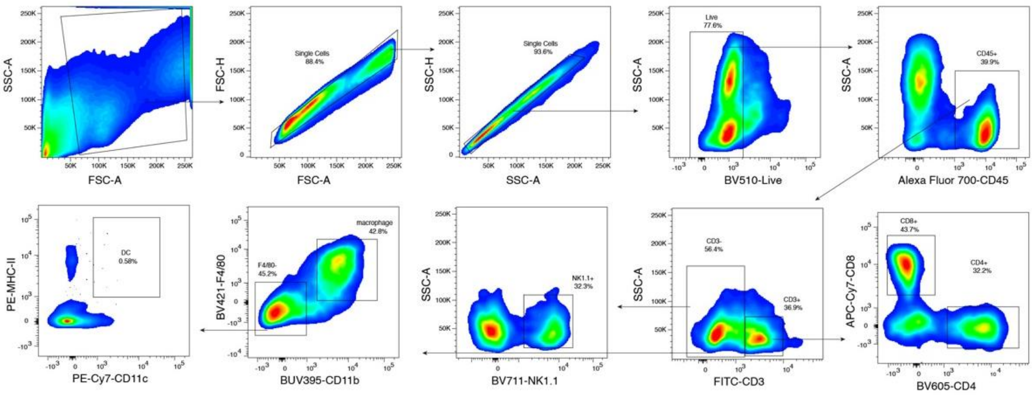

Supplementary Figure 2. Flow cytometry gating strategy of $CD45^{+}$ cells, $CD8^{+}$ T cells, $CD4^{+}$ T cells, NK cells, DC cells and macrophage.

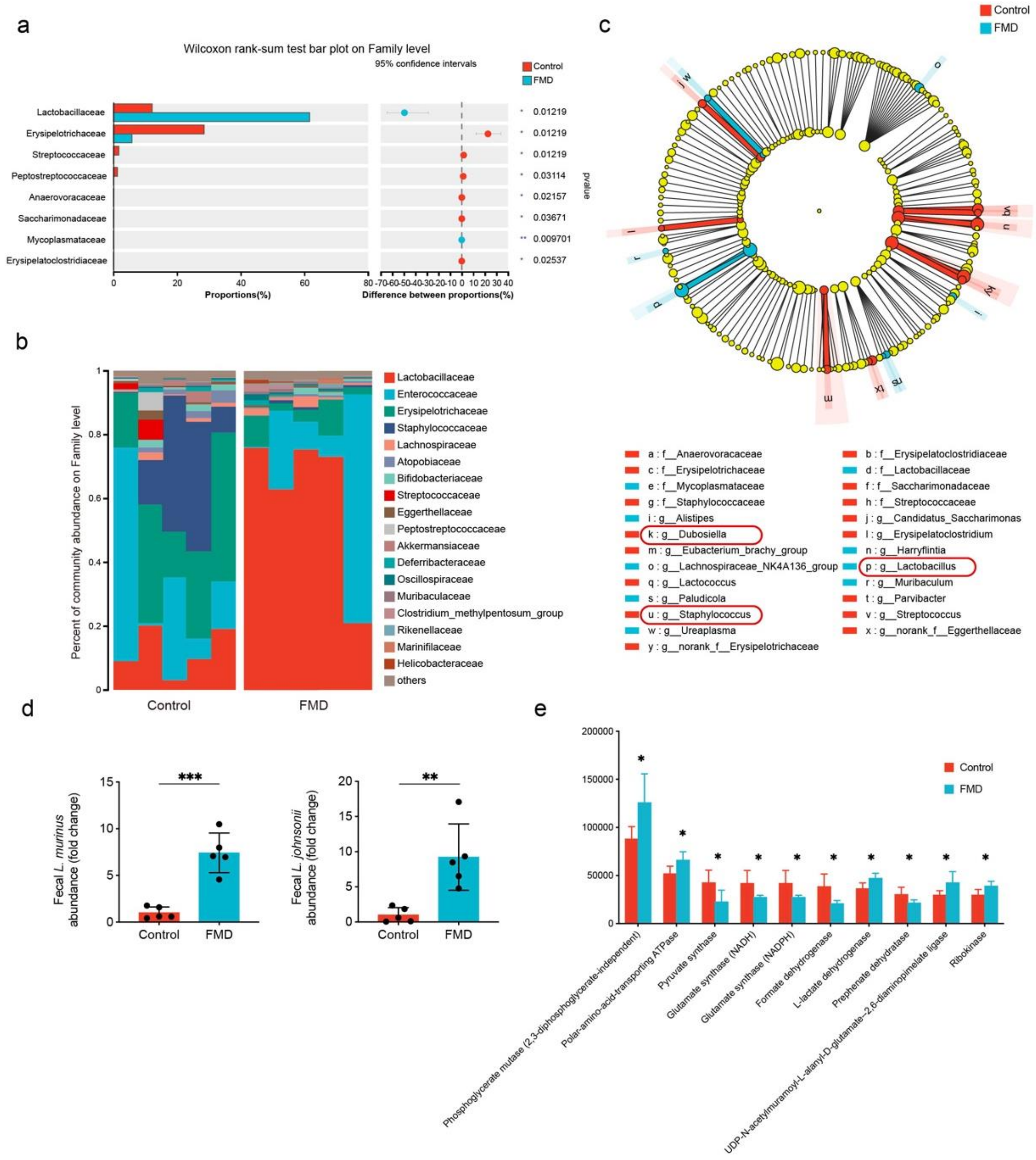

Supplementary Figure 3. FMD changed gut microbiota composition. **a.** Significant species alterations in control and FMD groups on family level. **b.** Plot summarizing the percentage community of abundance of microbial families in fecal samples from the control and FMD groups. **c.** LEfSe used to identify the differentially expressed bacterial taxa in two groups (family to genus level); the size and color of circles indicate the abundance of the bacterial. **d.** qPCR verification of fecal abundance of *L. johnsonii* and *L. murinus*. **e.** PICRUSt2 prediction of KEGG enzyme abundance from 16S rRNA, showing the top 10 most abundant enzymes. *p < 0.05, **p < 0.01, ***p < 0.001.

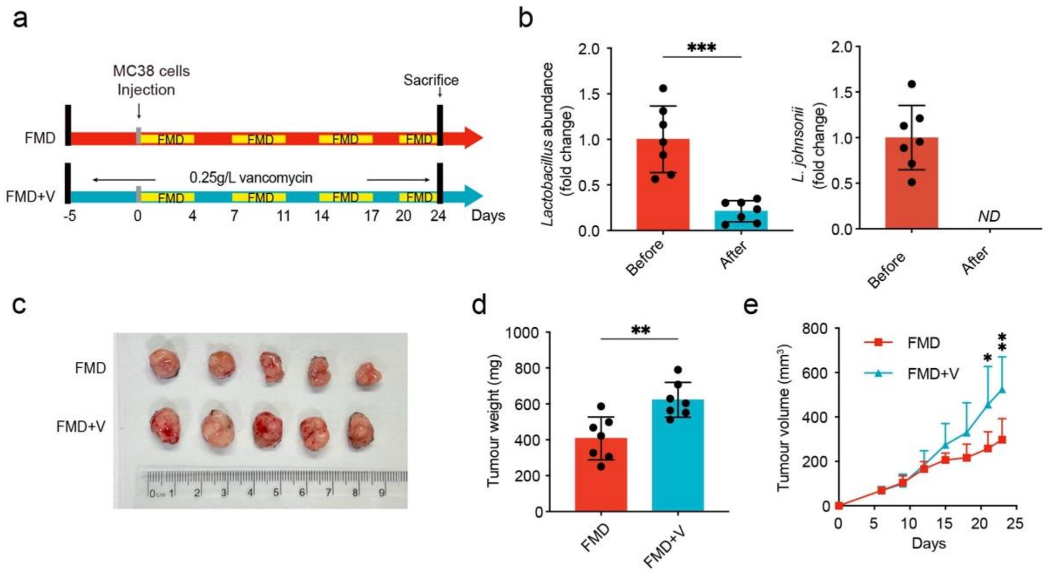

Supplementary Figure 4. The role of *Lactobacillus* and *L. johnsonii* in modulating tumor growth during FMD. **a.** Schematic diagram of FMD and vancomycin regimen. **b.** qPCR verification of fold change of *Lactobacillus* and *L. johnsonii* after vancomycin treatment before FMD started. **c.** MC38 cells were injected subcutaneously into C57BL/6 mice. Image of dissected tumors at day 24. Upper: FMD group; lower: FMD + vancomycin group. **d.** Tumor weight on day of sacrifice. **e.** Average tumor volume measured after MC38 cells injection. FMD group: n=7; FMD+V: n=7. Student t test. Data are shown in mean ± SD. *$p < 0.05$, **$p < 0.01$, ***$p < 0.001$.
